# Microwear and isotopic analyses on cave bear remains from Toll Cave reveal both short-term and long-term dietary habits

**DOI:** 10.1038/s41598-019-42152-7

**Published:** 2019-04-05

**Authors:** Iván Ramírez-Pedraza, Carlos Tornero, Spyridoula Pappa, Sahra Talamo, Domingo C. Salazar-García, Ruth Blasco, Jordi Rosell, Florent Rivals

**Affiliations:** 1 Institut Català de Paleoecologia Humana i Evolució Social (IPHES), Campus Sescelades URV (Edifici W3), 43007 Tarragona, Spain; 2 Universitat Rovira i Virgili (URV), Àrea de Prehistoria, Avinguda de Catalunya 35, 43002 Tarragona, Spain; 3 Department of Earth Sciences, Natural History Museum, Cromwell Road, London, SW7 5BD United Kingdom; 4 Department of Geography, Royal Holloway University of London, Egham, Surrey, TW20 0EX United Kingdom; 5 Department of Human Evolution, Max Planck Institute for Evolutionary Anthropology, Deutscher Platz 6, Leipzig, 04103 Germany; 6 Department of Geological Sciences, University of Cape Town, Cape Town, South Africa; 7 Grupo de Investigación en Prehistoria IT-622-13 (UPV-EHU)/IKERBASQUE-Basque Foundation for Science, Vitoria, Spain; 8 Centro Nacional de Investigación sobre la Evolución Humana (CENIEH), Paseo Sierra de Atapuerca 3, 09002 Burgos, Spain; 9 ICREA, Pg. Lluís Companys 23, 08010 Barcelona, Spain

## Abstract

Dietary habits of the extinct *Ursus spelaeus* have always been a controversial topic in paleontological studies. In this work, we investigate carbon and nitrogen values in the bone collagen and dental microwear of *U*. *spelaeus* specimens recovered in Level 4 from Toll Cave (Moià, Catalonia, NE Iberian Peninsula). These remains have been dated to > 49,000 ^14^C BP. The ability of both proxies to provide data on the diet of *U*. *spelaeus* at different times in the life-history (isotopes: average diet of life; microwear: last days/weeks before death), allows us to generate high-resolution and complementary data. Our results show lower values (δ^13^C & δ^15^N) in cave bears than in strict herbivores (i.e. *Cervus elaphus*) recovered from the same level of Toll Cave. On the other hand, 12 lower molars (m1) were analysed through low-magnification microwear technique. The cave bears from Toll Cave show a microwear pattern like that of extant bears with omnivorous and carnivorous diets. These data are discussed in the framework of all available data in Europe and add new information about the plasticity of the dietary habits of this species at the southern latitudes of Europe during Late Pleistocene periods.

## Introduction

The rapid climatic fluctuations that took place during the late Pleistocene led to repeated changes in both the environment and the vegetal landscape. These variations generated modifications in the biogeographical distribution of mammals in Europe^1–4^. The large carnivores are of importance when it comes to understanding the magnitude of these climatic alternations, because they occupy a large space and are less linked to particular biotopes as the herbivores^5,6^.

A paradigmatic example of this adaptive capacity is found in the cave bear (*U*. *spelaeus*), one of the most studied European members of the order Carnivora of the late Pleistocene. This species is a characteristic element of the last “Ice Age” and its remains have been found by the thousands in many European caves, such as the celebrated Drachenhöhle near Mixnitz in Styria (Austria)^7^. The geographical distribution of the cave bear group extends eastwards from northwest Spain across central Europe to the Urals, and from Belgium and the Harz region of Germany in the north to Italy and Greece in the south and to the Crimea in the southeast^8^. Among the key aspects related to the palaeoecology of this extinct animal are its feeding habits. Knowing the dietary habits of this species is essential for a better understanding of its durability and its biogeographical distribution across Europe. Such knowledge will allow us to go deeper into the factors that led to its extinction at the end of the late Pleistocene. Indeed, the topics of megafauna extinction during the late Pleistocene have been subject to intensive debates for decades and many of them are still valid today^8–11^. Some scientists have offered various hypotheses as to why there are important accumulations of this species in caves, one of which points to human activity: most notably, the cave bear was intensively hunted and may have been included in different rituals and cults^12^. However, other authors did not find clear evidence of these activities once the taphonomic origins of natural mass accumulations of this animal in caves were analysed^13–15^. In addition, investigations in several Alpine cave sites reported that cave bears and humans used particular caves at different times^14,15^. At present, there is still an important debate about the causes that could lead to the extinction of this species and about the possible role that human pressure or climatic changes could have played in this process^16–18^. Many authors suggested that the accumulations of *U*. *spelaeus* skeletal remains in caves are due to natural death of individuals, because they did not overcome the hibernation process, although the rate and timing of these accumulations remains poorly understood^19–23^. Something that also needs to be considered is that the disappearance of the cave bear from central Europe coincides fairly closely with the cooling climate and vegetation changes around the Last Glacial Maximum (LGM)^8^. Furthermore, as with all large animals, they existed in smaller populations than did small mammals and had a much slower reproduction rate, factors that counted against them^24^. Although interpreting the feeding ecology of Ursidae can be complex and difficult, nowadays it is possible to make inferences about cave bear feeding habits with the availability of innovative methods such as dental microwear and isotopic analyses.

The microwear studies allow us to explore the paleodiet of species and to reveal information about palaeoenvironmental changes^25–29^. In the Ursidae, this proxy began to be used relatively recently, using different methods^30–39^. Pinto-Lona^31,33^ compared the occlusal microwear and macrowear between *U*. *spelaeus* and *U*. *arctos*. They indicated that cave bears had a greater degree of bone consumption than did brown bears. Münzel *et al*.^35^ concluded that the predominance of pits over scratches is a typical pattern in herbivorous bears. On the other hand, Peigné *et al*.^32^ proposed a mixed diet for *U*. *spelaeus* and Jones and DeSantis^36^ suggested that *U*. *spelaeus* consumed a diet with a diversity of textural properties similar to most other bears and only distinguishable from the hyper-carnivorous polar bear (*Ursus maritimus*). Medin *et al*.^37^ suggested that the early Pleistocene *Ursus etruscus* bears from southern Spain were omnivorous with some consuming a significant amount of fish. Peigné and Merceron^38^ applied Dental Microwear Texture Analysis (DMTA) on cave bears from Belgium and their main conclusion was that, during the pre-dormancy period, these bears showed dietary flexibility and, most probably, excluded hard and brittle foods from their diet. Finally, more recently Pappa *et al*.^39^ developed a new comprehensive database of dental microwear features for extant Ursidae. The authors also proved that is possible to observe a differentiation of ecospaces within modern bear populations from different geographical regions. They then used this database to interpret the paleodiet in *U*. *arctos* from the late Middle Pleistocene site Grays Thurrock, U.K. This site demonstrated that these bears consumed mainly fibrous, soft food and invertebrates and a small vertebrate components.

Another useful technique is the analysis of stable isotopes. The publications made in this field concerning the feeding habits of the *U*. *spelaeus* show homogeneity in values, with results similar or inferior to those of contemporary herbivores of the same archaeological level. The low values of δ^15^N are purportedly linked to a predominantly vegetarian diet^40–55^. Conversely, Richards *et al*.^56^ and Robu *et al*.^57,58^ in Peştera cu Oase (Romania) show values of δ^15^N of the *U*. *spelaeus* that place it at the same level as contemporary carnivores, suggesting an omnivorous diet for this species. It is worth mentioning that decreasing δ^15^N values can result not only from reduced consumption of animal protein in the diet but also from variations in soil δ^15^N values due to climatic conditions linked with vegetation cover^50,59^ or by a higher amount of nitrogen-fixing plants in the animal’s diet. According to Fernández-Mosquera *et al*.^53^, δ^15^N values in nitrogen-fixing plants are lower than in plants that do not fix nitrogen. In addition, an analysis of bear blood revealed that the δ^13^C values during hibernation decrease, while the δ^15^N increases^60^. Hence, bear species have an interesting and complex metabolism (aspects of which remain poorly understood but which need to be considered when interpreting isotopic data).

The objective of this work is to approximate bear feeding habits in Mediterranean latitudes, providing two different and complementary temporal resolutions: the proxies of dental microwear and the stable isotopes. While the stable isotope analysis of δ^13^C and δ^15^N in the bulk-collagen of bone tissue samples provides an average information of the diet that the animal consumed during the last years prior to death^61^, microwear offers information of the diet that the animal ate during its last days/weeks before dying^62,63^.

A study of the feeding habits of *U*. *spelaeus* that combined stable isotopes extracted from bone collagen and dental microwear compared to a wide variety of extant species of ursids has never been published before. This work was performed on the fossil remains of cave bear from the Toll Cave (NE Spain) which is located at Mediterranean climate latitudes. Moreover, the cave bear remains and other bones from this site have been radiocarbon dated. It should also be noted that information on Mediterranean latitudes is practically non-existent, and these can be interesting when contributing data of the diet of this animal in more temperate environments.

## Site and Materials

**Figure 1:**
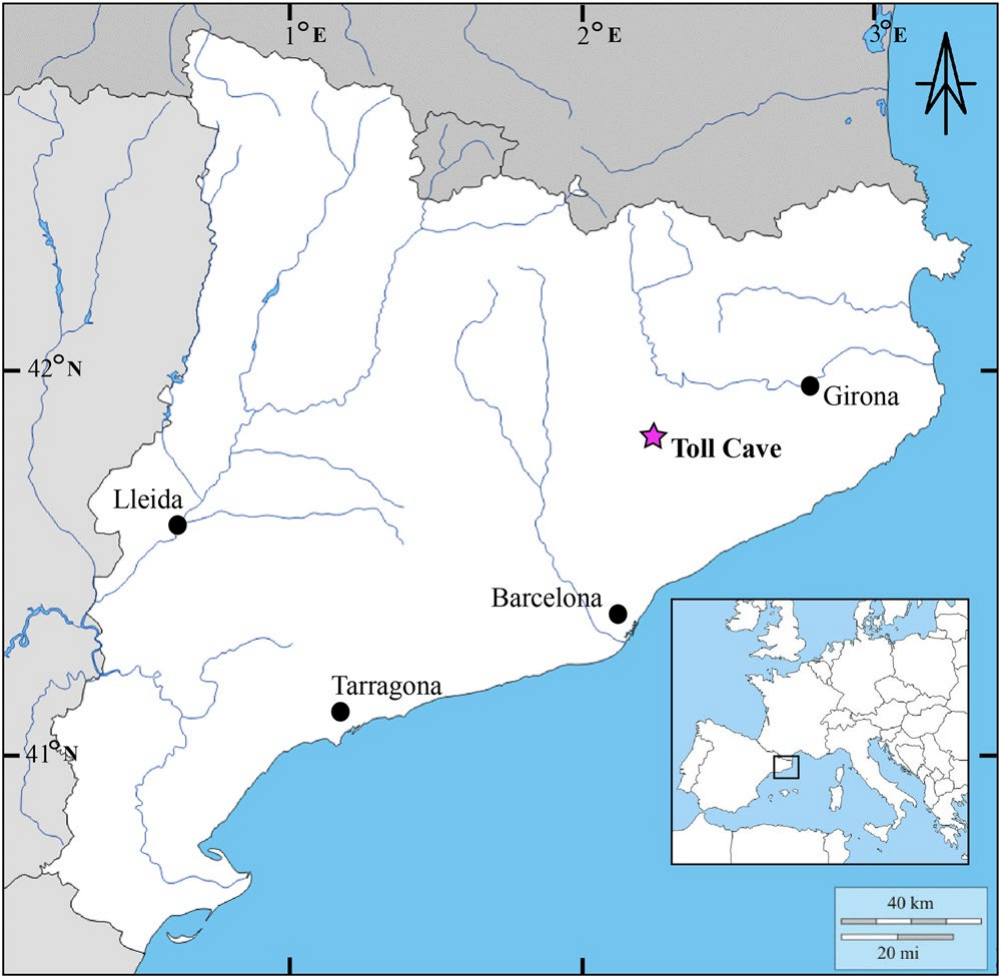
Map of the location of the site (Toll Cave, Spain).

Toll Cave is located near the village of Moià, 50 km to the north of Barcelona (Fig. 1). It is one of the caves belonging to a karstic system forming a course of galleries of more than 2 km long. The cave is at about 760 m a.s.l., and its coordinates are 2°09′02″ E and 41°48′25″ N. To date, four archaeological levels have been excavated. The Holocene sediments (level 1) show evidence of being mixed, level 2 is probably Holocene (<13 ka BP) and level 3 is late Pleistocene (>13 ka BP)^64^. Level 4 has been recently excavated and new radiocarbon dates are presented in this paper. In this work, all the faunal remains analysed come from Level 4. At this level, different species have been identified, mainly cave bear, but also carnivores such as spotted hyenas (*Crocuta crocuta*), lions (*Panthera leo spelaea)*, and wolves (*Canis lupus)*, as well as small carnivores such as lynxes (*Lynx pardina)*, wildcats (*Felis silvestris*), foxes (*Vulpes vulpes*) and badgers (*Meles meles*). There are also ungulates, such as rhinoceros (*Stephanorhinus* sp.), horses (*Equus ferus*), European asses (*Equus hydruntinus*), red deer (*Cervus elaphus*), roe deer (*Capreolus capreolus*), wild boar (*Sus scrofa*) and rabbits (*Oryctolagus cuniculus*). The assemblage has been identified as belonging to the Upper Pleistocene and interpreted as the result of a hibernation lair, especially intense in Level 4. This is supported by the abundance of remains of *U*. *spelaeus* and the taphonomic characteristics of the assemblage, where the activity of carnivores, such as hyenas and wolves, is significant^65–67^.

All the material available from the recent excavations has been considered for this work. The material is currently stored in the collections of the Catalan Institute of Human Paleoecology and Social Evolution (IPHES, Tarragona, Spain). At present, Toll Cave is one of the sites with one of the most important collections of this ursid fossil in the Iberian Peninsula^65–67^.

## Results

### Dental microwear analysis

Dental microwear analysis (DMA) performed on the samples of extant wild bears shows, in general, a total average of pits that is higher than the total average of scratches in all species (Table 1). The general pattern shows a higher number of fine scratches than coarse scratches for all species. *A*. *melanoleuca* has the highest number of fine scratches and *U*. *arctos* (Greece) has the highest number of coarse scratches. If we consider the scratch width (SWS), *A*. *melanoleuca* do not show any coarse and hypercoarse scratch, the rest of the species show a mixture of fine and coarse scratches and *U*. *maritimus* shows a predominance of hypercoarse scratches. Among the extant bear species, the average number of small pits is higher than the average number of large pits except for *U*. *arctos* from Greece. *A*. *melanoleuca* is the species with the highest average number of small pits. *U*. *arctos* (N. America) has the highest average number of scratches (NTS) and *U*. *maritimus* has the lowest average number of scratches (NTS), but the pattern is very similar among species. In the case of the total average number of pits (NTP), the most remarkable data is the higher number observed in *A*. *melanoleuca*, especially in small pits that double in number those of the other species.

**Table 1:** Comparison of the average DMA results between the molars of *U*. *spelaeus* of Toll Cave and samples of extant bears published by Pappa *et al*.^39^ and Pappa^88^.

Species	N	NFS	NCS	NTS	SWS	LP	SP	NTP
*A*. *melanoleuca*	4	19.25	0.00	19.25	0.00	8.50	46.25	54.75
*H*. *malayanus*	17	17.29	2.24	19.53	0.94	5.12	18.53	23.65
*M*. *ursinus*	4	12.75	3.25	16.00	1.00	9.75	20.25	30.00
*T*. *ornatus*	2	13.00	3.00	16.00	1.00	7.00	18.50	25.50
*U*. *americanus*	9	13.56	2.56	16.11	1.00	5.44	19.00	24.44
*U*. *maritimus*	14	11.00	3.21	14.21	2.79	4.50	16.21	20.71
*U*. *thibetanus*	6	14.33	3.33	17.67	1.00	4.50	15.33	19.83
*U*. *arctos*, Greece	4	13.00	7.00	20.00	1.00	9.25	8.50	17.75
*U*. *arctos*, central EU	10	17.50	3.40	20.90	1.00	5.40	22.50	27.90
*U*. *arctos*, N. America	8	18.25	3.00	21.25	1.00	6.75	18.38	25.13
*U*. *arctos*, Russia	23	16.22	3.83	20.04	1.09	6.96	19.78	26.74
*U*. *arctos*, N. Europe	9	15.78	3.78	19.56	1.00	6.44	23.33	29.78
***U***. ***spelaeus*** **(Toll Cave)**	12	20.04	6.58	26.63	1.00	4.83	20.67	25.50
***U***. ***spelaeus*** **(Toll Cave) SD**	—	4.59	2.25	4.32	—	2.49	2.31	3.75

N = number of specimens; NFS = number of fine scratches; NCS = number of coarse scratches; NTS = total number of scratches; SWS = scratches width score; NSP = number of small pits; NLP = number of large pits; NTP = total number of pits; SD = standard deviation.

In comparison to the extant bear species, the *U*. *spelaeus* from Toll Cave has the highest number of scratches, both fine and coarse. However, the number of pits fits in the range of the extant species (Table 1; Supplementary Table S1).

**Figure 2:**
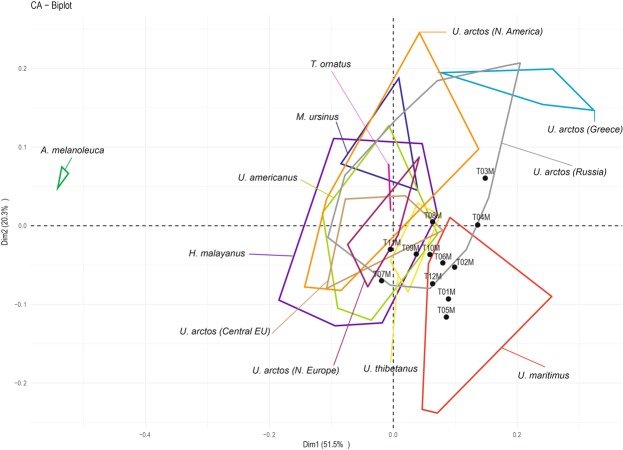
Correspondence Analysis (CA) based on five microwear variables (NFS = number of fine scratches; NCS = number of coarse scratches; SWS = scratches width score; NSP = number of small pits; NLP = number of large pits) for the extant ursid species and the cave bear from the Toll Cave.

A Correspondence Analysis (CA) was performed to compare all the microwear variables in the extant species and in the *U*. *spelaeus* from the Toll Cave. The results for axis 1 and 2 were plotted because its percentage of variance is higher than for the other axes (Table 2). The CA indicates that the polar bear (*U*. *maritimus*) is distant from the other species due to the presence of hypercoarse scratches (Fig. 2). The panda (*A*. *melanoleuca*) plots across axis 2 (respect to the other species) because it does not have any coarse and hypercoarse scratches and it is characterized by a high number of small pits. The *U*. *arctos* from Greece is in the upper right because it is the only species with a higher average of large pits than of small pits and because it has the highest percentage of coarse scratches. The specimens of *U*. *spelaeus* from Toll Cave are plotting far from the herbivorous species *A*. *melanoleuca* and the insectivorous species *M*. *ursinus*, as well as from *U*. *arctos* (Greece). The *U*. *spelaeus* appear near the omnivorous species *U*. *arctos* (Central Europe, Russia), *H*. *malayanus*, *U*. *thibetanus*, *U*. *americanus* and the carnivorous species *U*. *maritimus* (Fig. 2).

**Table 2:** Eigenvalues, variance percentages of each dimension (Dim.).

	Dim.1	Dim.2	Dim.3	Dim.4
Eigenvalues	0.018	0.007	0.005	0.005
% of var.	51.487	20.290	15.205	13.018
Cumulative % of var.	51.487	71.777	86.982	100.000

### Stable isotope analysis

#### Collagen preservation

The results of the stable isotopes and collagen quality indicators are reported in Table 3. Collagen was successfully extracted from 32 of 39 (82%) samples. It was not possible to extract collagen in five samples (Toll 9, 16, 23, 24, 38) because collagen yields were lower than 10 mg. g^−1^. Some samples (Toll 7, 10, 12, 13, 15, 22, 29–34, 36) present C and N contents (C% and N% values) lower than the recommended accepted limits in Van Klinken^68^ but higher than those suggested by Ambrose^69,70^. In these samples, the carbon and nitrogen % values were not correlated with the isotopic signatures (δ^13^C: Spearman’s rho, r = 0.294, p = 0.328; δ^15^N Spearman’s rho, r = −0.008, p = 0.978); rather in the C:N values, respectively (C%: Spearman’s rho, r = 0.666, p = 0.664; N% Spearman’s rho, r = 0.001, p = 0.996). In these samples, the absence of correlation within species was also verified. Two samples (Toll 27, 39) present C and N contents (C% and N% values) lower than recommended accepted limits in both Ambrose^69,70^ and Van Klinken^68^ proposals and were discarded for final interpretation. Using these criteria, and after seeing no correlation between atomic amount and isotope values in the remaining samples, we decided to include for interpretation all samples with a successful recovery of C% and N% in collagen range from 14 to 40% and from 5 to 14%, respectively, as well as with atomic C:N ratio ranging from 3.1 to 3.5 (mean value 3.3 ± 0.10).

**Table 3:** Results of δ^13^C and δ^15^N values and collagen preservation (C%, N% and C:N) of Toll Cave samples.

Sample	Taxa	Element	Side	Age category	Code	Yield (mg/g)	δ^13^C_V-PDB_ ‰	δ^15^N_AIR_ ‰	C %	N %	C:N
Toll Bovidae 13	*Bos* sp.	humerus	—	adult	P10/07	25	−22.0	3.1	17.1	6.2	3.2
Toll Bovidae 17	*Bos* sp.	radius	—	adult	P10/07	27	−19.6	6.6	32.8	11.7	3.3
Toll Bovidae 24	*Bos* sp.	astragalus	l	adult	P11/03	—	—	—	—	—	—
Toll Bovidae 25	*Bos* sp.	tibia	—	adult	Q07/3	34	−18.8	7.8	40.4	14.1	3.3
Toll Bovidae 6*	*Bos* sp.	radius	—	adult	P14/29	24	−20.7	4.1	39.7	14.1	3.3
Toll Bovidae 8	*Bos* sp.	tibia	l	adult	P11/01	33	−20.1	5.0	32.8	11.6	3.3
Toll Cervidae 12	*Cervus elaphus*	metatarsus	r	adult	Q17/32	32	−21.5	5.9	18.1	6.9	3.1
Toll Cervidae 2*	*Cervus elaphus*	femur	—	adult	Q10/08	33	−20.9	3.3	39.9	13.9	3.3
Toll Cervidae 3*	*Cervus elaphus*	tibia	—	adult	P13/52	49	−20.2	4.0	37.2	13.3	3.3
Toll Cervidae 39	*Cervus elaphus*	metapod	—	adult	Q10/50	27	−22.2	3.3	23.4	6.6	4.1
Toll Cervidae 4	*Cervus elaphus*	metapod	—	adult	Q17/38	30	−19.4	6.0	40.8	14.5	3.3
Toll Cervidae 9	*Cervus elaphus*	vertebrae	—	adult	R14/04	—	—	—	—	—	—
Toll Equidae 40	*Equus* sp.	metapod	—	adult	R12/45	35	−20.7	5.6	31.2	11.5	3.2
Toll Hyenidae 10	*Crocuta crocuta*	mandible	l	adult	P13/03	26	−19.7	9.5	26.9	9.8	3.2
Toll Hyenidae19*	*Crocuta crocuta*	metapod	—	adult	Q08/17	36	−18.7	10.0	31.4	11.6	3.2
Toll Rhinocerotidae 7	*Stephanorhinus* sp.	mandible	—	adult	P10/20	25	−18.9	5.9	24.8	8.6	3.4
Toll Ursidae 1*	*U*. *spelaeus*	femur	—	adult	Q10/13	28	−20.8	2.4	38.5	13.4	3.3
Toll Ursidae 14*	*U*. *spelaeus*	femur	r	immature	Q18/03	31	−22.9	8.2	38.6	12.7	3.5
Toll Ursidae 15	*U*. *spelaeus*	femur	l	adult	Q11/28	34	−21.0	2.8	30.3	10.8	3.3
Toll Ursidae 16	*U*. *spelaeus*	femur	r	adult	Q09/44	—	—	—	—	—	—
Toll Ursidae 18*	*U*. *spelaeus*	humerus	r	adult	P14/24	27	−20.4	2.3	36.0	13.5	3.1
Toll Ursidae 20*	*U*. *spelaeus*	femur	—	adult	P15/53	32	−20.2	6.7	33.6	12.7	3.1
Toll Ursidae 21*	*U*. *spelaeus*	femur	l	adult	Q10/48	31	−21.2	2.4	30.1	11.1	3.2
Toll Ursidae 22*	*U*. *spelaeus*	femur	—	sub—adult	Q11/44	35	−20.8	3.4	24.4	8.3	3.4
Toll Ursidae 23	*U*. *spelaeus*	femur	l	adult	Q09/59	—	—	—	—	—	—
Toll Ursidae 26	*U*. *spelaeus*	femur	r	adult	P10/25	26	−20.9	2.6	34.3	12.4	3.2
Toll Ursidae 27	*U*. *spelaeus*	mandible	r	adult	P12/23	40	−20.9	3.4	6.6	2.4	3.3
Toll Ursidae 28	*U*. *spelaeus*	mandible	r	adult	Q17/20	24	−21.2	2.7	37.8	13.5	3.3
Toll Ursidae 29	*U*. *spelaeus*	mandible	r	immature	P12/21	32	−20.4	2.9	29.0	10.8	3.1
Toll Ursidae 30	*U*. *spelaeus*	maxilla	r	adult	Q13/30	29	−20.4	5.3	31.3	10.9	3.3
Toll Ursidae 31	*U*. *spelaeus*	mandible	l	immature	P09/19	27	−22.0	5.0	27.7	9.9	3.3
Toll Ursidae 32	*U*. *spelaeus*	mandible	r	immature	Q10/45	26	−21.6	4.5	28.1	10.2	3.2
Toll Ursidae 33	*U*. *spelaeus*	humerus	l	sub-adult	Q08/11	33	−21.1	3.2	14.2	5.2	3.2
Toll Ursidae 34	*U*. *spelaeus*	humerus	r	adult	P17/1	25	−20.9	6.7	27.4	10.4	3.1
Toll Ursidae 35	*U*. *spelaeus*	humerus	l	immature	Q10/2	29	−21.1	3.8	35.3	13.0	3.2
Toll Ursidae 36	*U*. *spelaeus*	humerus	l	immature	Q10/9	27	−21.8	5.0	24.6	9.2	3.1
Toll Ursidae 37	*U*. *spelaeus*	humerus	r	adult	Q11/4	35	−20.9	2.6	30.1	11.1	3.2
Toll Ursidae 38	*U*. *spelaeus*	humerus	—	adult	Q12/41	—	—	—	—	—	—
Toll Ursidae 5*	*U*. *spelaeus*	femur	r	adult	Q09/37	32	−20.5	3.5	33.5	11.7	3.3

*Replicated samples in Cape Town laboratory.

#### δ^13^C and δ^15^N values

The δ^13^C values of all samples measured in the Toll Cave range from −22.9 to −18.7‰ (n = 32): in ursids, δ^13^C values range from −22.9 to −20.2‰ (n = 19); for the cervids, δ^13^C values range from −21.5 to −19.4‰ (n = 4); in bovids, δ^13^C values range from −22 to −18.8‰ (n = 5) and in the hyaenids, δ^13^C values range from −19.7 to −8.7‰ (n = 2). δ^13^C values in the unique sample of equids and rhinocerotids are −20.7‰ and −18.9‰, respectively.

The δ^15^N values of all samples measured in the Toll Cave range from 2.4 to 10‰ (n = 32): in ursids, δ^15^N values range from 2.4 to 8.2‰ (n = 19); in cervids, δ^15^N values range from 3.3 to 6‰ (n = 4); in bovids, δ^15^N values range from 3.1 to 7.8‰ (n = 5) and in the hyaenids, δ^15^N values range from 9.5 to 10‰ (n = 2). δ^15^N values in the unique sample of equids and rhinocerotids are 5.6‰ and 5.9‰, respectively.

**Figure 3:**
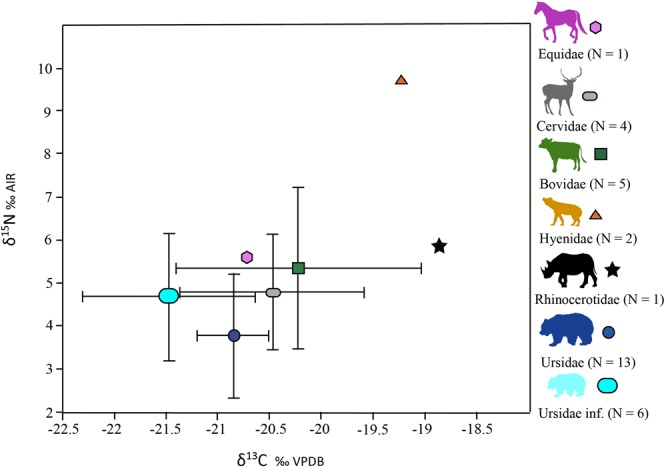
Average values of δ^13^C and δ^15^N of the different species analysed. The error bars correspond to the standard deviation.

Figure 3 shows the place occupied by each species in the trophic chain. In this case, the lowest nitrogen values correspond to the adult ursids and the highest correspond to the hyenas; all other herbivores and immature ursids are located between these two species. Carbon analysis place the rhino and hyena in the area that indicates more positive carbon values and the herbivores and ursids in the area with more negative values, given that the immature ursids have the most negative values.

Significant differences among all species and *U*. *spelaeus* were found only between Hyaenidae and adult and sub-adult cave bear groups of individuals (ANOVA and Tukey’s pairwise comparison: Q = 4.188 and p = 0.0357 for δ^13^C values; Q = 7.238 and p = 0.00028 for δ^15^N values). Statistical differences within cave bear samples were found between adult and sub-adult samples against immature specimens, but only in δ^13^C values (t*-*test; t = 3.1954; p = 0.0053).

### Comparison with other populations from the late pleistocene

**Figure 4:**
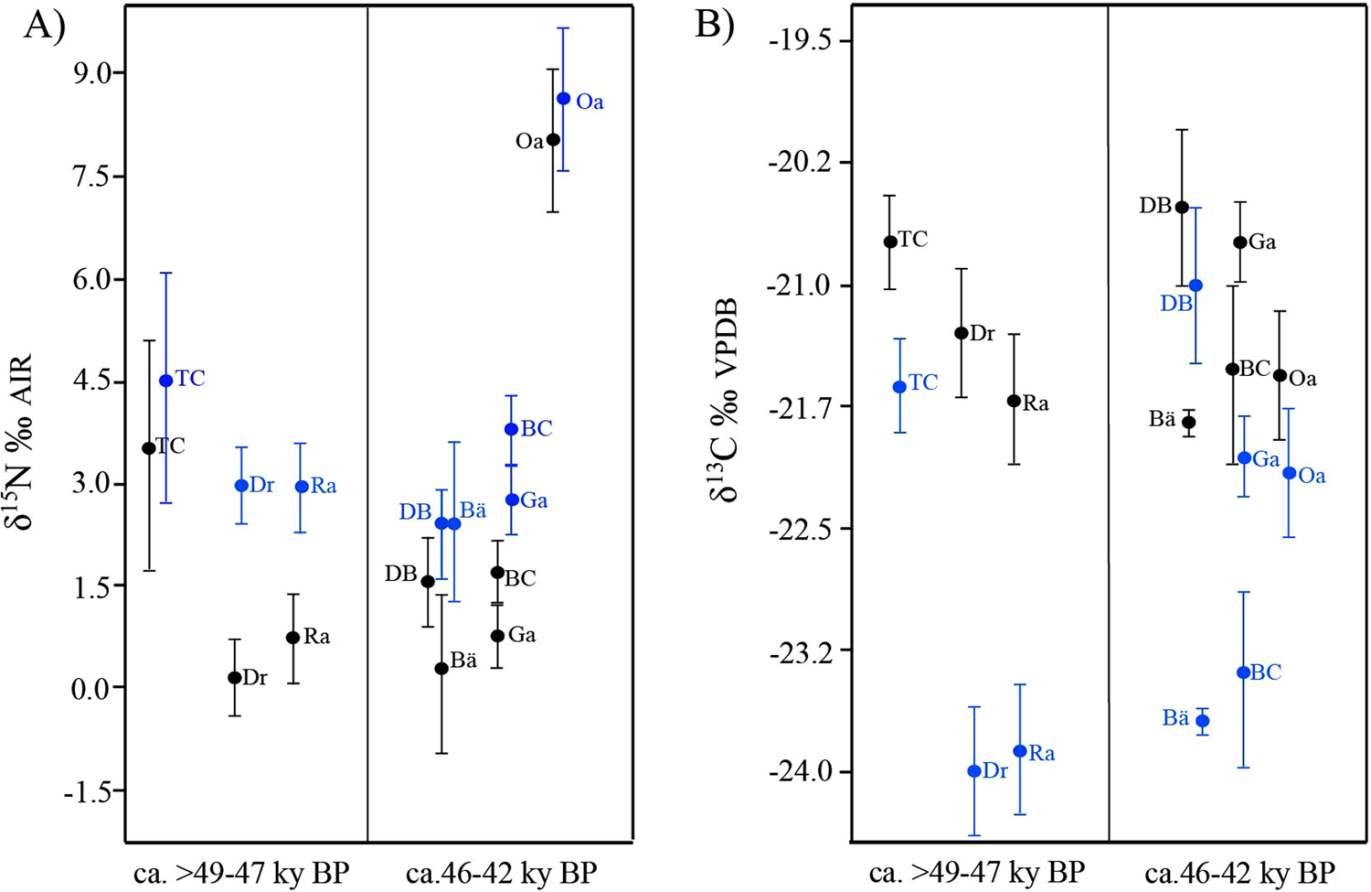
Comparison of the average values of δ^13^C and δ^15^N of the contemporaneous sites of Toll Cave, blue line with the altitudinal adjustment and black line without altitudinal adjustment. The error bars correspond to the standard deviation. (Selected sites are: TC = Toll Cave (Spain) 760 m; Oa = Peştera cu Oase (Romania) 600 m; Dr = Drachenloch (Switzerland) 2475 m; Bä = Bärenloch (Switzerland) 1645 m; Ra = Ramesch (Austria) 1960 m; BC = Balme à Collomb (France) 1700 m; DB = Divje Babe (Slovenia) 450 m; Ga = Gamssulzen (Austria) 1300 m).

In Fig. 4, we compare the isotopic results from the Toll Cave (TC) to all available isotopic data from contemporaneous (i.e., ^14^C dated) and cave bear specimens in Europe. Following the same approach as Krajcarz *et al*.^47^, we used the altitudinal adjustment published by Männel *et al*.^71^: δ^15^N-adj-alt = δ^15^N + (0.0011 · altitude), and δ^13^C-adj-alt = δ^13^C − (0.0011 · altitude), where altitude is given in meters. The correction removes the altitude bias and allows the equalizing of all data to the same level (i.e., 0 m a.s.l.) making the samples comparable. The statistical test with carbon adjustment shows significant differences between the Toll Cave and the sites of Drachenloch (Dr), Bärenloch (Bä), Ramesch (Ra) and Balme à Collomb (BC) (ANOVA and Tukey’s pairwise comparison: p-value for δ^13^C values, TC/Dr = 0.00014; TC/Bä = 0.00014; TC/Ra = 0.00014; TC/BC = 0.00018; TC/Ga = 0.6514; TC/DB = 0.5607). However, the statistical test with nitrogen adjustment does not show significant differences between the Toll Cave and the European selected sites (ANOVA and Tukey’s pairwise comparison: p-value for δ^15^N values, TC/Dr = 0.6074; TC/Bä = 0.0930; TC/Ra = 0.6074; TC/BC = 0.8452; TC/Ga = 0.1612; TC/DB = 0.0743). We excluded the Romanian site from the statistical test because its values were very different from the rest of European cave bear isotopic results.

### Radiocarbon

All four samples passed the quality criteria for radiocarbon dating proposed by Van Klinken^68^ (Table 4). Hence the extracted collagen were sent to the Mannheim AMS laboratory (Lab Code MAMS) for AMS dating^72^. All the ^14^C results were calibrated using the IntCal 13^73^ in IOxCal 4.3 program^74^. The results show that level 3 ranges between 46,660–45,900 cal BP at 68.2% probability, on the other hand, level 4 is quite old. The two Ursidae are dated outside the ^14^C range (>49,000 ^14^C BP). The only finite result is the bone of a large-sized mammal (MAMS - 18677; 47,310 ^14^C BP). When calibrated, this date is out of the range of radiocarbon arriving at max 49,860 cal BP at 68.2% probability. This is the only result that shows a slightly less amount of collagen (0.81%), when compared to all the other samples. However, this sample (S - EVA 27850) displays a normal C:N ratio. For this reason, we consider this date valid in its context.

**Table 4:** Radiocarbon dating, isotopic values, % of collagen and C:N ratios of dated samples from the Toll Cave.

MPI code number	Field number	Level	Taxa/ Bone	%Coll	δ^13^C	δ^15^N	%C	%N	C:N	AMS Nr.	^14^C Age	Err 1σ	Cal BP 68.2%	Cal BP 95.4%
S-EVA 27843	T09/P17/21	3	Cervidae/Tibia	1.95	−18.74	6.98	40.73	14.79	3.21	MAMS-18677	43130	340	46,660–45,900	47,140–45,580
S-EVA 27845	T11/Q12/60	4	Ursidae Mandible	4.07	−20.45	2.67	39.23	14.38	3.18	MAMS-18679	>49000			
S-EVA 27850*	T11/P16/71	4	Large size/flat bone	0.81	−20.81	3.76	36.18	13.27	3.18	MAMS-18684	47310	540	out of ^14^C range-49,860	out of ^14^C range-49,980
S-EVA 27851	T11/Q11/44	4	Ursidae/Femur	5.05	−19.89	3.98	40.22	14.76	3.18	MAMS-18685	>49000			

## Discussion

The average δ^13^C value in *U*. *spelaeus* (only adults) corresponds to animals with a dominant consumption of C_3_ plants. Carbon isotopes have a great potential for reconstructing past habitats and the δ^13^C values, which are more negative than in the rest of the herbivores and carnivores analysed, may be due to a more closed forest habitat for this species. This can be linked to the recycling of organic matter (canopy effect) impoverished in ^13^C that occurs in these dense forests^48,52,75^. Indeed, the δ^13^C values obtained in other species, related to open landscapes, show more positive carbon values. The more negative δ^13^C values of *U*. *spelaeus* compared to contemporary species are supported by the palaeoenvironmental analyses of the pollen record and the small mammals (including rodents) that were carried out in Toll Cave. Palynological results seem to show a closed forest environment with a predominance of *Pinus* sp. with the presence of some other taxa^76^. The analysis of the small mammal remains also supports the idea of a woodland habitat^64^. Alternatively, it has been suggested that lowest δ^13^C values in cave bear, in contrast with those of contemporary species, could be related to the storage of lipids during hibernation and their subsequent recycling in the synthesis process of some amino acids^41,48^. But if the carbon values of the Toll Cave ursids decreased during the hibernation process, nitrogen values should increase by the same process, as has been observed in modern ursids studies^60^. The latter is not documented in our results.

As for the δ^13^C values, the δ^15^N values obtained in the adult samples of *U*. *spelaeus* from Toll Cave are lower than those obtained in both contemporaneous carnivorous and herbivorous specimens sampled from the same archaeological level. This data suggests a lower position in the trophic chain and would indicate a mainly herbivorous diet for our bear specimens. However, these low values could also be explained by the fact that biological fixation of nitrogen causes a ^15^N decrease in the tissue of nitrogen-fixing plants in relation to those that do not fix it^53^. In this fixation process, atmospheric nitrogen is enzymatically converted to organic nitrogen, including amino acids, nucleotides and other molecules^77^. The fixation can be caused by several factors, such as the symbiosis of nodules in the roots of several plants (e.g. the Fabaceae family) with some bacteria, the non-symbiotic fixation carried out by an aerobic bacterium, or the rain^78^. Therefore, these low values of δ^15^N could be due to a preferential feeding of the *U*. *spelaeus* on nitrogen fixing plants, which include not only the Fabaceae family but a large taxonomic variety of plants from 8 families and 23 genera^79^. Moreover, a cave bear diet based on the Fabaceae family plants could be undetectable by δ^13^C values in our study (i.e. bulk collagen samples). The results of the pollen analysis at Toll Cave do not support this hypothesis because the pollen spectrum does not show the presence of nitrogen-fixing taxa. However, it must be taken into account that some taxa may appear underrepresented in the pollen record, due to their mode of dispersion and differential conservation, among other factors^80,81^.

The δ^13^C and δ^15^N values of the immature bears at Toll Cave are different from those of the adult bears. Although statistical differences are only attested in carbon values between immature and adults, nitrogen values show a trend toward higher values in immature specimens. Isotopic values of young mammals may have been influenced by the metabolic changes of the mother during pregnancy, such as the breastfeeding and the hibernation process. The offspring usually have higher values of δ^15^N due to lactation and the proteins in milk^82^. During hibernation, the tissues of the immature bear are formed from the metabolic process derived from the fat storage of the mother. The recycling of the mother’s nitrogen during the gestation of the foetus could result in elevated δ^15^N values in its tissues^23^. The depleted δ^13^C values observed in immature bears could be explained as the result of ^12^C from the mother’s fat being incorporated into the collagen of immature bears preferentially. Triglycerides are the main constituents of body fat and these are composed of glycerol and three fatty acids. Glycerol from triglycerides metabolism enters the glycolytic pathway and the carboxyl carbons of amino acids arising from glycolytic intermediates would be especially depleted in ^13^C^61^.

As tooth microwear reflects the diet of the last days/weeks before death, this analysis offers the opportunity to characterize the diet to which the *U*. *spelaeus* had access at a specific time in its life. Our results show that the diet of the cave bear may not always have been that of a strictly vegetarian animal because they have a microwear pattern like that of extant omnivorous and carnivorous species. Since the isotopic analyses do not record the short-term diet, it is not possible to know the seasonal dynamics of *U*. *spelaeus* from this proxy. However, microwear results suggest a varied and less specialized diet before the death of these individuals, indicating a dietary plasticity that implies that the cave bear had the capacity to adapt to the availability of resources due to factors such as seasonal changes. Considering the amount of energy and body mass that a bear of such size must acquire in order to successfully overcome the period of hibernation, it would make sense not to adopt a strictly herbivorous diet before hibernating^83^. This situation does not occur in winter or spring, where cases of extant bears, such as grizzlies, adopting a strictly vegetarian diet have been documented^84,85^. One interesting hypothesis, defended by some authors^19–23^, considers that many of the remains of *U*. *spelaeus* found in caves belong to individuals who died during the hibernation process, an important seasonal event in the life of these animals. Considering this hypothesis, microwear analysis makes it possible to establish the bear feeding habits before their hibernation.

The isotopic signatures from the cave bear specimens of the Toll Cave, which are older than 49,000 years BP, were compared to data available in other published studies. The results are similar to the values registered for *U*. *spelaeus* in most of the European sites on which isotope analysis has been carried out, with the exception of the works published by Richards *et al*.^56^ and Robu *et al*.^57,58^ in Peştera cu Oase (Romania), which show values of δ^15^N of the *U*. *spelaeus* that place it at the same level of contemporary carnivores. The values registered everywhere else indicate so far an herbivorous diet for *U*. *spelaeus*^35,41,47,48,50,51,53,86–88^.

Nevertheless, the comparison between isotopic data from different cave bears around Europe must be carried out by taking into account the effect of some external factors on both the isotopic carbon and nitrogen signatures. For instance, in Krajcarz *et al*.^47^, the authors demonstrate that altitudinal location affects significantly the fixation of δ^13^C and δ^15^N signatures, while latitude does not show any apparent effects. No differences at the latitudinal level may be because the altitudinal range of the sites analysed in Europe is very narrow. However, the differences at the altitudinal level are remarkable. In our comparison, the lowest altitude site is Divje Babe (Slovenia) 450 m a.s.l. and the highest altitude is Drachenloch (Switzerland) 2475 m a.s.l.

The differences in carbon values is more related to the altitudinal position than to the resources. Our results show differences in carbon between Toll Cave (TC) and the sites located at elevations above 1500 m a.s.l. as Drachenloch (Dr), Bärenloch (Bä), Ramesch (Ra) and Balme à Collomb (BC). This means that ursid populations fed on plants with a different carbon signal. Photosynthetic groups of plants that could show a different δ^13^C signature or also that these animals inhabited landscapes with a varied plant cover (canopy effects)^47^.

On the contrary, the lack of difference in the isotopic nitrogen signatures between populations that are likely to be contemporaneous in Europe but located at different altitudes suggests that probably the differences between soils were not very significant and the contribution of protein in cave bear diet was minimal in all sampled population (except in Peştera cu Oase, not considered for comparison). In the Toll Cave, the low position of the cave bear in the trophic chain, similar or lower than in likely contemporaneous strict herbivores, indicates a low protein intake in its diet. The same pattern is observed in other European sites^23,88^, including Drachenloch, Bärenloch, Ramesch and Balme à Collomb sites^44^.

## Conclusion

The integrated analysis of stable isotopes and microwear allowed us to confirm the significance of these two proxies for studies focused on paleodiet. The ability of both proxies to provide data on the diet of *U*. *spelaeus* at different timeframes (average diet of life vs. last days/weeks before death) allows for the generation of more complete and complementary results that provide better consistency in this type of palaeodietary studies. According to the isotopic values, the diet of the *U*. *spelaeus* located in the Mediterranean area at the Toll Cave is like that of other European sites of the Late Pleistocene. We did detect some differences in the δ^13^C values that are probably related to the vegetal landscape of different sites located at different altitudes. It shows a mostly herbivorous lifetime diet. Considering the homogeneity in δ^15^N values (except for the Romanian sites), other significant factors beyond the diet such as human pressure can be contemplated as the cause of the extinction of this ursid. However, tooth microwear patterns of the cave bear are like those of extant omnivorous and carnivorous ursid species, suggesting dietary flexibility and abilities to shift towards a more omnivorous diet. Our results show the usefulness of the integration of these two proxies for providing information on the cave bear’s diet at different times of its life.

In future works, it will be interesting to study the isotopic signal of the same extant ursid specimens that were used to create the microwear database. At the same time, this type of multi-proxy study will be performed in other sites with different chronologies. Special attention will be given to chronologies close to the extinction of the cave bear in order to observe if a common microwear pattern exists in the same way that is observed for isotopic values.

## Methods

### Dental microwear analysis (DMA)

Dental microwear analysis was performed using the light stereomicroscopy technique established by Solounias and Semprebon^27^. This technique was selected because a large reference database that includes all bear species has been recent published^39^. A total of 12 first lower molars of *U*. *spelaeus* (m1, carnassial) were selected for the (DMA). All teeth showed an occlusal surface wear indicative of prime adults at the time of death (categories IV, V, VI and VII of Stiner^20^). The minimum number of individuals (MNI) is equal to 12, considering the laterality and the size of the teeth as well as the degree of wear. Enamel microwear features were observed via standard light stereomicroscopy at x35 magnification on high-resolution epoxy casts of teeth, following the cleaning, moulding, casting and examination protocol developed by Solounias and Semprebon^27^ and Semprebon *et al*.^89^. The occlusal surface of each specimen was cleaned using acetone and then 96% ethanol. Once dry, the moulding substance, a high-resolution dental silicone (i.e. vinylpolysiloxane) suitable for microwear analysis, was applied with a gun directly on to the tooth and casts were created using transparent epoxy resin. Before the final selection of 12 molars, the teeth with bad preservation or other taphonomical marks were excluded from the subsequent analysis^90^. These casts were examined using a Zeiss Stemi 2000C stereomicroscope at low magnification. A standard 0.4 × 0.4 = 0.16 mm^2^ ocular reticle was employed to quantify the number of small and large pits (round scars), scratches (elongated scars with parallel sides), scratch width score (a score of zero (0) is given when only fine scratches are present, one (1) when there is a mixture of fine and coarse scratches on the surface, two (2) when predominantly coarse scratches are present and three (3) when the surface has also hypercoarse scratches). In carnivores, the facets of the slicing and grinding areas are usually examined. However, for our study we focus on non-faceted enamel surfaces because they are more decisive in the comparison between different species of ursids. Primates and bears have multicusped premolars and molars and tooth morphology, and belong to the omnivorous group^91^. In this sense, and considering our focus on bears, it is adequate to use unworn surfaces without facets rather than worn surfaces of the tooth^39,92^. The results have been compared with the new reference databased on extant bears established by Pappa *et al*.^39^, which includes the following species and even brown bear specimens from different geographical latitudes: *Ursus arctos* (Brown bear) from Greece, central and north of Europe, N. America and Russia, *Ursus maritimus* (Polar bear), *Ursus americanus* (Black bear), *Ailuropoda melanoleuca* (Giant panda), *Ursus thibetanus* (Asian black bear), *Helarctos malayanus* (Sun bear), *Melursus ursinus* (Sloth bear) and *Tremarctos ornatus* (Spectacled bear).

### Collagen extraction and isotope analyses

A total of 23 remains of cave bear were selected and sampled for stable carbon and nitrogen isotope analysis. These correspond to a total of 11 different individuals (8 adults, 1 sub-adult and 2 immature) considering taxonomical identification by osteological criteria, estimated age and bilateral symmetry. To define the local baseline of the trophic food chain for the ursid palaeodietary reconstruction, we also selected a range of contemporaneous carnivores and ungulates (Hyenidae n = 2, Bovidae n = 6, *Cervus elaphus* n = 6, Equidae n = 1 and Rhinocerotidae n = 1), all recovered in Level 4.

Collagen extraction was performed at the Biomolecular Laboratory of IPHES in Tarragona (Spain). For each specimen, a small bone fragment was carefully sawed with a Dremmel rotating tool equipped with a circular diamond-coated blade, ultrasonicated in acetone and water, rinsed with distilled water, dried and crushed to a powder of < 0.7 mm grain size. The collagen was purified according to Login’s acid-base-acid protocol published in 1971^93^, subsequently modified in Bocherens *et al*.^94^. Bone shards (ca. 300 to 350 mg) were soaked in 1 M HCl for demineralization, in NaOH (0.125 M) to remove contaminants, rinsed with distilled water, and gelatinized with 0.01 M HCl at 100 °C for 17 h. Once filtered and frozen, samples were freeze dried at the Institute of Chemical Research in Catalonia (ICIQ). Gelatine-collagen samples weighing about 300 μg were analysed using a Thermo Flash 1112 elemental analyser (EA) coupled to a Thermo Delta V Advantage isotope ratio mass spectrometer (IRMS) with a Conflo III interface, at the Institute of Environmental Science and Technology (ICTA), Autonomous University of Barcelona (Barcelona, Spain). The international standard laboratory IAEA 600 (caffeine) was used as control. The average analytical error was <0.15‰ (1σ) calculated for each of the isotopic measures, δ^13^C and δ^15^N, separately. Some collagen samples of ca. 0.500 mg were also analysed at the Stable Light Isotope Laboratory of the University of Cape Town (South Africa) in duplicate using a Thermo Flash EA 1112 interfaced with a Delta plus XP. Samples analysed between both labs had a standard deviation of (1σ) <0.1. The reliability of the isotopic signature of the collagen extracts was assessed using several criteria in both laboratories (yield of extraction; percentages of C% and N%; and the atomic C/N ratio). We have assumed a range of atomic C:N ration between 2.9 to 3.6 as indicator of good preservation of collagen^68–70,95^. Isotope ratios are expressed for carbon as δ^13^C Vienna Pee Dee Belemnite (V-PDB) and for nitrogen as δ^15^N atmospheric nitrogen (AIR): d X ¼ (R_sample_/R_standard_ − 1) * 1000‰, where **χ** stands for ^13^C or ^15^N and R stands for ^13^C/^12^C or ^15^N/^14^N.

### Radiocarbon pre-treatment

Four bone samples from Toll Cave were pre-treated for radiocarbon dating at the Department of Human Evolution at the Max Planck Institute for Evolutionary Anthropology (MPI-EVA), Leipzig, Germany, using the method described in Talamo and Richard^96^. The outer surface of the bone samples is first cleaned by a shot blaster and then 500 mg of the whole bone is taken. The samples are then decalcified in 0.5 M HCl at room temperature for about 4 hours or until no CO_2_ effervescence is observed. To remove humic acids, 0.1 M NaOH is added for 30 minutes. The NaOH step is followed by a final 0.5 M HCl step for 15 minutes. The resulting solid is gelatinized following Longin^93^ at pH3 in a heater block at 75 °C for 20 h. The gelatine is then filtered in an Eeze-Filter™ (Elkay Laboratory Products (UK) Ltd.) to remove small ( > 80μm) particles. The gelatine is then ultrafiltered^97^ with Sartorius “VivaspinTurbo” 30 KDa ultrafilters. Prior to use, the filter is cleaned to remove carbon containing humectants^98^. The samples are lyophilized for 48 hours.

In order to monitor contamination introduced during the pre-treatment stage, a sample from a cave bear bone, kindly provided by D. Döppes (MAMS, Germany), was extracted along with the batch from the human specimen^99^.

### Statistics

The bivariate graphs and the statistics t-test and the ANOVA and Tukey’s pairwise comparison were made with the software Past 3.15^100^. The significance of p-values was fixed <0.05. The correspondence analysis was performed using the package ca (v. 0.70) in R language^101^. The script was adapted from the STHDA-statistical tools for high-throughput data analysis (sthda.com).

## Supplementary information

Supplementary Table S1

## Data Availability

All data generated during this study are included here and in the Supplementary Information file.

## References

[1] Hofreiter M, Stewart J (2009). Ecological Change, Range Fluctuations and Population Dynamics during the Pleistocene. Curr. Biol..

[2] Kjellström E (2010). Simulated climate conditions in Europe during the Marine Isotope Stage 3 stadial. Boreas.

[3] Fletcher WJ (2010). Millennial-scale variability during the last glacial in vegetation records from Europe. Quat. Sci. Rev..

[4] Stuart AJ, Lister AM (2012). Extinction chronology of the woolly rhinoceros *Coelodonta antiquitatis* in the context of late Quaternary megafaunal extinctions in northern Eurasia. Quat. Sci. Rev..

[5] Ewer, R. F. *The Carnivores*. *The World Naturalist*. (Cornell University Press, 1973).

[6] Turner, A. & Antón, M. *The Big Cats and their Fossil Relatives*. (Columbia University Press, 1997).

[7] Kahlke, D. *The History of the Origin*, *Evolution and Dispersal of the Late Pleistocene Mammuthus-Coelodonta Faunal Complex in Eurasia (Large Mammals)*. (Fenske Companies, 1999).

[8] Pacher M, Stuart AJ (2009). Extinction chronology and palaeobiology of the cave bear (*Ursus spelaeus*). Boreas.

[9] Martin, R. Les mammifères fossiles du gisement quaternaire de Villereversure. Etude des Carnivores, des Cervidés et des Equidés. (PhD Thesis, Université de Lyon, 1968).

[10] Koch PL, Barnosky AD (2006). Late Quaternary Extinctions: State of the Debate. Annu. Rev. Ecol. Evol. Syst..

[11] Baca, M. *et al*. Retreat and extinction of the Late Pleistocene cave bear (*Ursus spelaeus* sensu lato). *Sci*. *Nat*. **103**, (2016). 10.1007/s00114-016-1414-8 PMC5059403 27730265

[12] Bächler E (1921). Das Drachenloch ob Veattis im Taminatale, 2445 m ü. M. und seine Bedeutung als paleaontologische Fundstatte und preahistorische Niederlassung aus der Altsteinzeit (Paleaolithikum) im Schweizerlande. Separatabdruck, Jahrb. St. Gall. Naturwissenschaftlichen Gesellschaft.

[13] Soergel, W. *Das Massenvorkommen des Höhlenbären* (1940).

[14] Pacher, M. Höhlenbäre und Mensch: Tatsachen und Vermutungen. in *Der* Höhlenbär (eds Rabeder, G., Nagel, D. & Pacher, M.) **4**, 82–104 (Species, Jan Thorbecke Verlag 2000).

[15] Pacher, M. Taphonomic analyses of cave bear remains from Potoèka zijalka (Slovenia): Further analyses and conclusion. In *Potoèka zijalka - Paleontological and archaeological result of the campaigns 1997–2000* (eds Pacher, M., Pohar, V. & Rabeder, G.) **13**, 97–114 (Mitteilungen der Kommission für Quartärforschung der Österreichischen Akademie der Wissenschaften, 2004).

[16] Sandom, C., Faurby, S., Sandel, B. & Svenning, J. C. Global late Quaternary megafauna extinctions linked to humans, not climate change. *Proc*. *R*. *Soc*. *B Biol*. *Sci*. **281** (2014). 10.1098/rspb.2013.3254 PMC4071532 24898370

[17] Stuart AJ (2015). Late Quaternary megafaunal extinctions on the continents: A short review. Geol. J..

[18] Terlato G (2019). Chronological and Isotopic data support a revision for the timing of cave bear extinction in Mediterranean Europe. Hist. Biol..

[19] Andrews P, Turner A (1992). Life and death of the Westbury bears. Ann. Zool. Fennici.

[20] Stiner MC (1998). Mortality analysis of Pleistocene bears and its paleoanthropological relevance. J. Hum. Evol..

[21] Pinto-Llona, A. C., Andrews, P. J. & Etxebarria, F. *Taphonomy and Palaeoecology of Bears from the Quaternary of Cantabrian Spain*. (Fundación oso de Asturias, 2005).

[22] Wojtal, P. *Zooarchaeological Studies of the Late Pleistocene Sites in Poland*. (Polish academy of sciences. Institute of systematics and evolution of animals, 2007).

[23] Grandal-d’Anglade A, Pérez-Rama M, García-Vázquez A, González-Fortes GM (2019). The cave bear’s hibernation: reconstructing the physiology and behaviour of an extinct animal. Hist. Biol..

[24] Lister, A. M. & Bahn, P. *Mammoths*. *Giants of the Ice Age*. (University of California Press, 2007).

[25] Walker A, Hoeck HN, Perez L (1978). Microwear of mammalian teeth as an indicator of diet. Science..

[26] Teaford MF, Walker A (1984). Quantitative differences in dental microwear between primate species with different diets and a comment on the presumed diet of *Sivapithecus*. Am. J. Phys. Anthropol..

[27] Solounias N, Semprebon GM (2002). Advances in the Reconstruction of Ungulate Ecomorphology with Application to Early Fossil Equids. Am. Museum Novit..

[28] Rivals F, Semprebon GM (2011). Dietary plasticity in ungulates: Insight from tooth microwear analysis. Quat. Int..

[29] Rivals F, Lister AM (2016). Dietary flexibility and niche partitioning of large herbivores through the Pleistocene of Britain. Quat. Sci. Rev..

[30] Pinto Llona AC, Andrews PJ (2001). Dental wear and grit ingestion in extant and extinct bears from Northern Spain. Cuad. do Lab. Xeol. Laxe.

[31] Pinto Llona, A. C. Comparative dental microwear analysis of Cave Bears *Ursus spelaeus* Rosenmüller, 1794 and Brown Bears *Ursus arctos* Linnaeus, 1758. *Sci*. *Ann*. *Sch*. *Geol*. *Aristotle Univ*. *Thessaloniki* **98**, 103–108 (2006).

[32] Peigne S (2009). Predormancy omnivory in European cave bears evidenced by a dental microwear analysis of Ursus spelaeus from Goyet, Belgium. Proc. Natl. Acad. Sci..

[33] Pinto-Llona AC (2013). Macrowear and occlusal microwear on teeth of cave bears *Ursus spelaeus* and brown bears *Ursus arctos*: Inferences concerning diet. Palaeogeogr. Palaeoclimatol. Palaeoecol..

[34] Donohue, S. L., DeSantis, L. R. G., Schubert, B. W. & Ungar, P. S. Was the giant short-faced bear a hyper-scavenger? A new approach to the dietary study of ursids using dental microwear textures. *PLoS One* **8** (2013). 10.1371/journal.pone.0077531 PMC3813673 24204860

[35] Münzel SC (2014). Behavioural ecology of Late Pleistocene bears (*Ursus spelaeus*, *Ursus ingressus*): Insight from stable isotopes (C, N, O) and tooth microwear. Quat. Int..

[36] Jones, D. B. & DeSantis, L. Dietary ecology of the extinct cave bear (*Ursus spelaeus*): evidence of omnivory as inferred from dental microwear textures. *Acta Palaeontol*. *Pol*. **61**, (2016).

[37] Medin T, Martínez-Navarro B, Rivals F, Libsekal Y, Rook L (2015). The late Early Pleistocene suid remains from the paleoanthropological site of Buia (Eritrea): Systematics, biochronology and eco-geographical context. Palaeogeogr. Palaeoclimatol. Palaeoecol..

[38] Peigné S, Merceron G (2019). Palaeoecology of cave bears as evidenced by dental wear analysis: a review of methods and recent findings. Hist. Biol..

[39] Pappa S, Schreve DC, Rivals F (2019). The bear necessities: A new dental microwear database for the interpretation of palaeodiet in fossil Ursidae. Palaeogeogr. Palaeoclimatol. Palaeoecol..

[40] Martin JE, Tacail T, Balter V (2017). Non-traditional isotope perspectives in vertebrate palaeobiology. Palaeontology.

[41] Bocherens H, Fizet M, Mariotti A (1994). Diet, physiology and ecology of fossil mammals as inferred from stable carbon and nitrogen isotope biogeochemistry: implications for Pleistocene bears. Palaeogeogr. Palaeoclimatol. Palaeoecol..

[42] Grandal-d’Anglade, A., Pérez-Rama, M. & Fernández-Mosquera, D. Diet, physiology and environment of the cave bear: a biogeochemical study. In *Fragments of Ice Age Environments*. *Proceedings in Honour of Ivan Turk’s Jubilee* (ed. Toskan, B.) 111–125, 10.13140/RG.2.1.3732.9445 (Inštitut za arheologijo ZRC SAZU, Založba ZRC, 2011).

[43] Münzel SC (2011). Pleistocene bears in the Swabian Jura (Germany): Genetic replacement, ecological displacement, extinctions and survival. Quat. Int..

[44] Pacher, M., Bocherens, H., Döppes, D., Frischauf, C. & Rabeder, G. First results of stable isotopes from Drachenloch and Wildenmannlisloch, Swiss Alps. in *Proceedings of the 17th International Cave Bear Symposium*, *15th-18th September 2011* (eds Döppes, D., Joger, U. & Rosendahl, W.) **11**, 101–110 (Braunschweiger Naturkundliche Schriften, 2012).

[45] Bocherens H, Grandal-d’Anglade A, Hobson KA (2014). Pitfalls in comparing modern hair and fossil bone collagen C and N isotopic data to reconstruct ancient diets: A case study with cave bears (*Ursus spelaeus*). Isotopes Environ. Health Stud..

[46] Naito YI (2016). Evidence for herbivorous cave bears (*Ursus spelaeus*) in Goyet Cave, Belgium: implications for palaeodietary reconstruction of fossil bears using amino acid δ^15^N approaches. J. Quat. Sci..

[47] Krajcarz M (2016). Isotopic variability of cave bears (δ^15^N, δ^13^C) across Europe during MIS 3. Quat. Sci. Rev..

[48] Bocherens H (1997). Paleobiological Implications of the Isotopic Signatures (^13^C, ^15^N) of Fossil Mammal Collagen in Scladina Cave (Sclayn, Belgium). Quat. Res..

[49] Bocherens, H. Cave bear palaeoecology and stable isotopes: checking the rules of the game. in *Proceedings of the 9th International* Cave Bear *Conference* (eds Philippe, M., Argant, A. & Argant, J.) 183–188 (Cahiers scientifiques du Centre de Conservation et d’Etude des Collections. Museum d’Histoire naturelle de Lyon, 2004).

[50] Bocherens H, Drucker DG, Billiou D, Geneste JM, van der Plicht J (2006). Bears and humans in Chauvet Cave (Vallon-Pont-d’Arc, Ardèche, France): Insights from stable isotopes and radiocarbon dating of bone collagen. J. Hum. Evol..

[51] Bocherens H (2015). Isotopic tracking of large carnivore palaeoecology in the mammoth steppe. Quat. Sci. Rev..

[52] Fernandez Mosquera D (1998). Biogeoquimica isotopica (δ^13^C, δ^15^N) del *Ursus spelaeus* del yacimiento de Cova Eiros, Lugo. Cad. do Lab. Xeol. Laxe.

[53] Fernández-Mosquera D, Vila-Taboada M, Grandal-d’Anglade A (2001). Stable isotopes data (δ^13^C, δ^15^N) from the cave bear (*Ursus spelaeus*): A new approach to its palaeoenvironment and dormancy. Proc. R. Soc. B Biol. Sci..

[54] Bocherens, H. Isotopic biogeochemistry and the paleoecology of the mammoth steppe fauna. In *ADVANCES IN MAMMOTH RESEARCH (Proceedings of the Second International Mammoth Conference)* (eds Reumer, J. W. F., De Vos, J. & Mol, D.) **1000**, 57–76 (2003).

[55] Bocherens H (2004). Diet reconstruction of ancient brown bears (*Ursus arctos*) from Mont Ventoux (France) using bone collagen stable isotope biogeochemistry (^13^C, ^15^N). Can. J. Zool..

[56] Richards MP (2008). Isotopic evidence for omnivory among European cave bears: Late Pleistocene *Ursus spelaeus* from the Peştera cu Oase, Romania. Proc. Natl. Acad. Sci. USA.

[57] Robu M (2013). Isotopic evidence for dietary flexibility among European Late Pleistocene cave bears (*Ursus spelaeus*). Can. J. Zool..

[58] Robu M (2017). The diverse dietary profiles of MIS 3 cave bears from the Romanian Carpathians: insights from stable isotope (δ^13^C and δ^15^N) analysis. Palaeontology.

[59] Rosendahl W, Grupe G (2001). Mittelwürmzeitliche Höhlenbären und ihre Nahrungspräferenz - Forschungen aus der Neuen Laubenstein-Bärenhöhle/Chiemgau. Mitt.Bayer.Staatss.Paläont.hist.Geol..

[60] Lohuis TD, Harlow HJ, Beck TDI (2007). Hibernating black bears (*Ursus americanus*) experience skeletal muscle protein balance during winter anorexia. Comp. Biochem. Physiol. - B Biochem. Mol. Biol..

[61] Keeling CI, Nelson DE (2001). Changes in the intramolecular stable carbon isotope ratios with age of the European cave bear (*Ursus spelaeus*). Oecologia.

[62] Grine FE (1986). Dental evidence for dietary differences in *Australopithecus* and *Paranthropus*: a quantitative analysis of permanent molar microwear. J. Hum. Evol..

[63] Teaford MF, Oyen OJ (1989). Differences in the Rate of Molar Wear between Monkeys Raised on Different Diets. J. Dent. Res..

[64] Fernández-García M, López-García JM (2013). Palaeoecology and biochronology based on the rodents analysis from the Late Pleistocene/Holocene of Toll Cave (Moià, Barcelona). Spanish J. Palaeontol..

[65] Rosell, J. *et al*. Els óssos hibernen, els neandertals passen: Noves dades sobre els desplaçaments per la Catalunya Central dels grups humans del Paleolític mig a partir dels resultats de les coves del Toll i de les Teixoneres (Moià, Bages). In *Actes III Jornades d’Arqueologia de la Catalunya Central 2014* (ed. Blasco, M. A.) 95–99 (Generalitat de Catalunya, Departament de Cultura, 2015).

[66] Rosell, J. *et al*. Compartint l’espai: la interacció entre homínids i carnívors al nord-est peninsular (Cova del Toll i Cova de les Teixoneres, Moià, Bages). In *Actes I Jornades d’Arqueologia de La Catalunya Central 2010* (eds Argemí, M. *et al*.) 47–51 (Generalitat de Catalunya, Departament de Cultura, 2012).

[67] Rosell, J. *et al*. Cova del Toll y Cova de les Teixoneres, Moià, Barcelona. in *Los cazadores recolectores del Pleistoceno y del Holoceno en Iberia y el Estrecho de Gibraltar: Estado actual del conocimiento del registro arqueológico* (ed. Sala Ramos, R.) 302–307 (Universidad de Burgos, 2014).

[68] Van Klinken GJ (1999). Bone collagen quality indicators for palaeodietary and radiocarbon measurements. J. Archaeol. Sci..

[69] Ambrose SH (1990). Preparation and characterization of bone and tooth collagen for isotopic analysis. J. Archaeol. Sci..

[70] Ambrose, S. H. & Norr, L. Experimental evidence for the relationship of the carbon isotope ratios of whole diet and dietary protein to those of bone collagen and carbonate. in *Prehistoric Human Bone: Archaeology at the Molecular Level* (eds Lambert, J. B. & Grupe, G.) 1–37, 10.1007/978-3-662-02894-0 (Springer-Verlag, 1993).

[71] Männel TT, Auerswald K, Schnyder H (2007). Altitudinal gradients of grassland carbon and nitrogen isotope composition are recorded in the hair of grazers. Glob. Ecol. Biogeogr..

[72] Kromer B, Lindauer S, Synal HA, Wacker L (2013). MAMS - A new AMS facility at the Curt-Engelhorn-Centre for Achaeometry, Mannheim, Germany. Nucl. Instruments Methods Phys. Res. Sect. B Beam Interact. with Mater. Atoms.

[73] Reimer P (2013). IntCal13 and Marine13 Radiocarbon Age Calibration Curves 0–50,000 Years cal BP. Radiocarbon.

[74] Ramsey CB (2009). Bayesian Analysis of Radiocarbon dates. Radiocarbon.

[75] Ambrose SH, DeNiro MJ (1989). Climate and habitat reconstruction using stable carbon and nitrogen isotope ratios of collagen in prehistoric herbivore teeth from Kenya. Quat. Res..

[76] Allué, E. *et al*. Cova del Toll (Moià, Bages): Perspectiva paleoambiental i arqueobotànica del Plistocè i Holocè. *Quad*. *Prehistòria Catalana* 21–38 (2013).

[77] Koch, P. L., Fogel, M. L. & Tuross, N. Tracing the diets of fossil animals using stable isotopes. In *Stable isotopes in ecology and environmental Science* (eds Lajtha, K. & Michener, R.) 63–92 (Blackwell Scientific Publications, 1994).

[78] Strasburger, E. *et al*. *Tratado de botánica*. (OMEGA, 1994).

[79] Salisbury, F. B. & Ross, C. W. *Plant Physiology*, *Hormones and Plant Regulators: Auxins and Gibberellins*. (Wadsworth Publishing, 1992).

[80] Faegri, K. & Iversen, J. *Textbook of Pollen Analysis*. (John Wiley and Sons, 1989).

[81] Moore, P. D., Webb, J. A. & Collinson, M. E. *Pollen analysis*. (Wiley-Blackwell, 1991).

[82] Fogel, M., Tuross, N. & Owsley, D. Nitrogen isotope tracers of human lactation in modern and archeological populations. *Annu*. *Rep*. *Dir*. *Nutr*. *Lab*. Carnegie Inst. *Washingt*. 111–117 (1989).

[83] Christiansen P (1999). What size were *Arctodus simus* and *Ursus spelaeus* (Carnivora: Ursidae)?. Ann. Zool. Fennici.

[84] Bull EL, Torgersen. T, Wertz TL (2001). The lmportance of Vegetation, Insects, and Neonate Ungulates in Black Bear Diet in Northeastern Oregon. Northwest Sci..

[85] Rode KD, Robbins CT, Shipley LA (2001). Constraints on herbivory by grizzly bears. Oecologia.

[86] Bocherens, H., Germonpre, M., Toussaint, M. & Semal, P. Stable isotopes, Spy Cave: State of 125 Years of Pluridisciplinary Research on the Betche-aux-Rotches from Spy. In *Spy Cave: State of 125 Years of Pluridisciplinary Research on the Betche-aux-Rotches from* Spy (eds Semal, P. & Hauzeur, A.) **1**, 357–371 (Butlletin de la Société royale belge d’Anthropologie et de Préhistoire, 2014).

[87] Pérez-Rama M, Fernández-Mosquera D, Grandal-d’Anglade A (2011). Recognizing growth patterns and maternal strategies in extinct species using stable isotopes: The case of the cave bear *Ursus spelaeus* ROSENMÜLLER. Quat. Int..

[88] Bocherens H (2019). Isotopic insights on cave bear palaeodiet. Hist. Biol..

[89] Semprebon GM, Godfrey LR, Solounias N, Sutherland MR, Jungers WL (2004). Can low-magnification stereomicroscopy reveal diet?. J. Hum. Evol..

[90] King T, Andrews P, Boz B (1999). Effect of Taphonomic Processes on Dental Microwear. Am. J. Phys. Anthropol..

[91] Hillson, S. *Teeth*, 10.3390/ht6040016 (Cambridge University Press, 2005).

[92] Pappa, S. Palaeoecology and Palaeodiet: reconstructing adaptations in the Middle and Late Pleistocene Ursidae through dental microwear and geochemistry. (PhD Thesis, Royal Holloway University of London, 2016).

[93] Longin R (1971). New method of collagen extraction for radiocarbon dating. Nature.

[94] Bocherens H (1991). Isotopic Biogeochemistry (δ^13^C, δ^15^N) of fossil vertebrate collagen: implications for the study of fossil food web including Neandertal Man. J. Hum. Evol..

[95] DeNiro MJ (1985). Postmortem preservation and alteration of *in-vivo* bone collagen isotope ratios in relation to palaeodietary reconstruction. Nature.

[96] Talamo S, Richards M (2011). A Comparison of Bone Pretreatment Methods for AMS Dating of Samples >30,000 BP. Radiocarbon.

[97] Brown TA, Nelson DE, Vogel JS, Southon JR (1988). Improved Collagen Extraction by Modified Longin Method. Radiocarbon.

[98] Brock F, Ramsey CB, Higham T (2007). Quality assurance of ultrafiltered bone dating. Radiocarbon.

[99] Korlević P, Talamo S, Meyer M (2018). A combined method for DNA analysis and radiocarbon dating from a single sample. Sci. Rep..

[100] Hammer Ø, Harper DA, Ryan PD (2001). Past: Paleontological Statistics Software Package for Education and Data Analysis. Palaeontol. Electron..

[101] R Core Team. *A language and environment for statistical computing*. *R Foundation for Statistical Computing* (2017).

