# Supplementary material for: Microwear and isotopic analyses on cave bear remains from Toll Cave reveal both short-term and long-term dietary habits
Source: Sci Rep. 2019 Apr 5;9:5716. doi: 10.1038/s41598-019-42152-7 (PMC6450970; doi:10.1038/s41598-019-42152-7)
Supplement: Supplementary file 1 — Supplementary Table S1 [file 41598_2019_42152_MOESM1_ESM.pdf]

Supplementary information

Microwear and isotopic analyses on cave bear remains from Toll Cave reveal both short-term and long-term dietary habits

Iván Ramírez-Pedraza[1,2, *], Carlos Tornero[1,2], Spyridoula Pappa[3,4], Sahra Talamo[5], Domingo C. Salazar-García[5],[6,7], Ruth Blasco[8], Jordi Rosell[1,2], Florent Rivals[1,2,9]

(1) Institut Català de Paleoecologia Humana i Evolució Social (IPHES), Campus Sescelades URV (Edifici W3), 43007 Tarragona, Spain

(2) Área de Prehistoria, Universitat Rovira i Virgili (URV), Avinguda de Catalunya 35, 43002 Tarragona, Spain

(3) Department of Earth Sciences, Natural History Museum, Cromwell Road, London SW7 5BD, United Kingdom

(4) Department of Geography, Royal Holloway University of London, Egham, Surrey TW20 0EX, United Kingdom

(5) Department of Human Evolution, Max Planck Institute for Evolutionary Anthropology, Deutscher Platz 6, Leipzig, 04103, Germany

(6) Department of Geological Sciences, University of Cape Town, Cape Town, South Africa

(7) Grupo de Investigación en Prehistoria IT-622-13 (UPV-EHU)/IKERBASQUE-Basque Foundation for Science, Vitoria, Spain

(8) Centro Nacional de Investigación sobre la Evolución Humana (CENIEH), Paseo Sierra de Atapuerca 3, 09002 Burgos, Spain

(9) ICREA, Pg. Lluís Companys 23, 08010 Barcelona, Spain

***Corresponding author:** I. Ramírez-Pedraza (ivan680@msn.com)

Supplementary Table 1. Tooth microwear raw data of the cave bear (*Ursus spelaeus*) from the Toll Cave. NFS = number of fine scratches; NCS = number of coarse scratches; NTS = total number of scratches; SWS = scratches width score; NSP = number of small pits; NLP = number of large pits; NTP = total number of pits.

Toll Cave	NSF	NSC	NST	NPS	NPL	NPT	SWS
**T01**	15	6	21	4.5	3	7.5	1
**T02**	19.5	6	25.5	3	2.5	5.5	1
**T03**	16	5.5	21.5	3	4	7	1
**T04**	21.5	3.5	25	5	4	9.5	1
**T05**	21	6	27	4	6	10	1
**T06**	23	5.5	28.5	5.5	5	10.5	1
**T07**	24.5	6.5	31	5.5	5	10.5	1
**T08**	24	4.5	28.5	7	5.5	12.5	1
**T09**	22	7	29	8	4	12	1
**T10**	23	8.5	31.5	7	4.5	11.5	1
**T11**	16	10.5	26.5	7.5	8.5	16	1
**T12**	15	9.5	24.5	8	6	14	1
